# Levels of anti-oxidative molecules and inflammatory factors in patients with vascular dementia and their clinical significance

**DOI:** 10.12669/pjms.37.5.3854

**Published:** 2021

**Authors:** Fan-xing Qi, Ying Hu, Ya-wei Li, Juan Gao

**Affiliations:** 1 Fan-xing Qi Department of Neurology, Baoding First Central Hospital, Baoding 071000, Hebei, China; 2 Ying Hu Department of Cardiology, Baoding First Central Hospital, Baoding 071000, Hebei, China; 3 Ya-wei Li Department of Medical Record Management, Baoding First Central Hospital, Baoding 071000, Hebei, China; 4 Juan Gao Department of Neurology, Baoding First Central Hospital, Baoding 071000, Hebei, China

**Keywords:** Vascular dementia, inflammatory factor, anti-oxidative factor

## Abstract

**Objectives:**

To explore levels of anti-oxidative molecules and inflammatory factors in patients with vascular dementia (VD) and their clinical significance.

**Methods:**

Sixty VD patients admitted in our hospital from January 2016 to January 2019 were classified into an experimental group, while another 60 healthy patients seeking physical examinations in the corresponding period were selected as a control group. Various indexes related to serum inflammatory factors and anti-oxidative molecules were compared among patients in such two groups. For the purpose of comparing anti-oxidative molecular expression levels and inflammatory factor levels in patients with VD of different severities, 60 cases in the experimental group were divided, based on a Mini-mental State Examination (MMSE) scale, into patients with mild symptoms (n=20, score: 21~26), patients with moderate symptoms (n=22, score: 10~20) and patients with severe symptoms (n=18, score: 0~9).

**Results:**

By contrast to the control group, levels of inflammatory factors (e.g., TNF-a, CRP and IL-6) in VD patients are all significantly increased and their differences show statistical significance (p<0.05); and, expression levels of anti-oxidative factors, including superoxide dismutase (SOD), glutathion peroxidase (GSH-Px), total antioxidant capacity (TAC), catalase (CAT) and glutathione reductase (GR), in the experimental group are apparently below those of the control group (P<0.05). As dementia degree increases, expression levels of serum anti-oxidative molecules in such patients are inclined to drop in a significant way (P<0.05), while inflammatory factor levels tend to go up gradually (P<0.05).

**Conclusions:**

If compared with the normal population, inflammatory factor levels in serum of VD patients are higher; however, expression levels of anti-oxidative molecules become below those of the normal population. Additionally, levels of inflammatory factors and anti-oxidative molecules may change obviously as severity of illness increases. This suggests that inflammation and oxidation play a certain role of auxoaction in VD patients.

## INTRODUCTION

Vascular dementia is the most common complication in elderly patients with cerebrovascular ischemia and hemorrhagic cerebrovascular diseases.^1^ Poor blood supply to the brain caused by vasculopathy can lead to brain hypoxia and also a series of pathologic changes such as hypoperfusion and inflammatory responses. This may finally give rise to cognitive dysfunction of brain.^2^ As pointed out in relevant studies, both ischemia reperfusion injury and oxidation-antioxidation imbalance after the injury are important causes of VD development.^3^ In the present study, not only is comparative analysis made on differences in inflammatory factors and anti-oxidative molecules in VD patients and the normal population compared, but their expression levels in VD patients with diverse severities of illness are also comparatively analyzed. It is indicated by comparison results that inflammatory factors and anti-oxidative molecules in VD patients are significantly different from those of the normal population. Moreover, such differences may become increasingly apparent as the severity of illness increases. For details of the present study, they are described as follows.

## METHODS

Sixty VD patients hospitalized in Baoding First Central Hospital from January 2016 to January 2019 were selected as the experimental group, while another 60 healthy cases seeking physical examinations in the corresponding period were adopted as the control group. To be specific, the experimental group consists of 37 male and 23 female patients 62~74 years old (average age: 69.26±3.07). In the control group, there are 34 male and 26 female cases 60~77 years old (average age: 69.58±5.07). As shown by comparison among general data of patients in both groups, no statistical significance is proved (p>0.05). This signifies that their general data are of comparability.

### Ethical Approval

The study was approved by the Institutional Ethics Committee of Baoding First Central Hospital on January 10, 2019, and written informed consent was obtained from all participants

### Inclusion criteria:

1. Patients meeting diagnostic criteria of VD^4^.
2. Patients with cerebral infarction or cerebral hemorrhage as suggested by head CT or nuclear magnetism.
3. Patients without cognitive dysfunction before occurrence of strokes.

### Exclusion criteria:

1. Patients with diseases affecting levels of inflammatory factors and anti-oxidative factors, such as chronic inflammatory diseases, autoimmune diseases and malignant tumors.
2. Patients with diseases related to thrombus at other sites, such as pulmonary embolism, myocardial infarction and lower extremity vascular embolization.
3. Patients with other diseases causing cognitive dysfunction.
4. Patients taking antipsychotics recently and thus suffering cognitive or mental dysfunction.

All patients selected and the normal population in the control group agreed with the present study protocol and signed the informed consent.

The MMSE scale was utilized to evaluate dementia severity of 60 patients from the experimental group. Based on three ranges of scoring, that is, 21~26, 10~20 and 0~9, the experimental group was further divided into three teams of 20, 22 and 18 patients showing mild, moderate and severe symptoms respectively.

Totally, 120 patients were included in experimental and control groups. In the morning, venous blood was drawn from them with empty stomach. Here, serum inflammatory factors tested consisted of TNF-α, IL-6 (ELISA; and, the assay kit is provided by R& D Systems, France) and CRP (turbidimetric immunoassay; and, the kit was provided by Roche). In addition, anti-oxidative factors were also examined, such as SOD, GSH-Px, TAC, CAT and GR, by radioimmunoassay (the kit was provided by Elabscience). After that, differences in above indexes were comparatively analyzed for experimental and control groups. Furthermore, levels of serum inflammatory factors and anti-oxidative molecules were assessed specific to patients at diverse dementia degrees.

### Statistical analysis

All data are enumerated by SPSS 20.0 and corresponding measurement data are denoted by ±S. While analysis on data obtained from experimental and control groups is made by independent-samples T test, correlation of indexes in the experimental group to dementia degrees is expressed in Pearson’s correlation coefficient. In case of P<0.05, differences are statistically significant.

## RESULTS

As shown by comparative analysis on serum inflammatory factors (Table-I), levels of serum TNF-a, CRP and IL-6 in VD patients from the experimental group are all significantly higher than those of the control group. Their differences are of statistical significance, that is, P<0.05.

**Table-I:** Target comparison for inflammatory factors in experimental and control groups in (X̅ ±S; n=60).

Group	TNF-a(ng/L)*	CRP(mg/L) *	IL-6(ng/L) *
Experimental	47.5±13.8	3.47±0.55	15.10±5.23
Control	24.3±9.2	1.22±0.71	8.97±2.24
t	10.84	19.41	8.35
p	0.00	0.00	0.00

* P<0.05.

According to results of comparative analysis on anti-oxidative molecules in experimental and control groups, as shown in Table-II, levels of serum SOD, TAC and CAT in VD patients are all significantly lower than those of the control group. Statistical significance is found in their differences (P<0.05). Regarding differences in GSH-Px and GR levels between both groups, no significant differences exist.

**Table-II:** Comparative analysis on anti-oxidative molecules in experimental and control groups (U/ml, n=60, X̅ ±S).

Group	SOD*	GSH-Px	TAC*	CAT*	GR
Experimental	62.18±7.13	337.39±29.76	12.02±1.77	8.14±1.06	126.53±11.32
Control	85.31±7.32	340.13±31.27	17.88±2.25	13.76±2.02	128.45±17.27
t	17.53	0.49	15.86	19.08	0.72
p	0.00	0.62	0.00	0.00	0.47

* P<0.05.

As manifested in Tables-III and IV, an increase in dementia degree corresponds to a gradual rise of serum inflammatory factor levels; that is, levels of serum inflammatory factors are positively correlated with severity of illness. More particularly, the maximum correlation is generated by TNF-a (p=0.00), which is followed by IL-6 (p=0.02); and, CRP is least correlated (p=0.04). With regards anti-oxidative molecules, levels of SOD, TCA and CAT gradually decline as severity of illness increases. This signifies that levels of anti-oxidative molecules have a negative correlation with dementia degree. Among these molecules, SOD and TAC are most negatively correlated (p<0.01), and a poor correlation is found in CAT (p<0.05). As for GSH-Px and GR, they are insignificantly correlated with severity of illness (p>0.05).

**Table-III:** Analysis on correlation of serum inflammatory factor targets and dementia degrees (r)

Dementia degree	n	TNF-a(ng/L)*	CRP(mg/L)#	IL-6(ng/L)#
Mild	20	0.133	0.136	0.031
Moderate	22	0.227	0.139	0.044
Severe	18	0.261	0.218	0.060
r		1.23	0.84	0.87
p		0.00	0.04	0.02

Bilateral significance p<0.05 (*p<0.01, #p<0.05).

**Table-IV:** Analysis on correlation of serum anti-oxidative molecule targets and dementia degrees (r).

Dementia degree	SOD*	GSH-Px	TAC*	CAT#	GR
Mild	-0.205	-0.251	-0.140	-0.145	-0.120
Moderate	-0.135	-0.203	-0.137	-0.137	-0.124
Severe	-0.072	-0.226	-0.125	-0.132	-0.118
r	1.47	0.03	1.12	0.79	0.08
p	0.00	0.67	0.00	0.03	0.32

Bilateral significance,

* p<0.01; # p<0.05.

## DISCUSSIONS

VD is a common disease among elderly patients. It is secondary to cerebral vascular accidents, including ischemic cerebrovascular diseases and hemorrhagic cerebrovascular diseases. Moreover, as chronic cerebral ischemia can lead to functional disorder of cerebral cells, it is deemed as a major cause of VD occurrence and development.^5^ Since occurrence of cerebral ischemia or anoxia, cerebral atrophy or dysfunction may be induced at diverse severities. In this aspect, inflammatory responses and oxidation-antioxidation imbalance are most critical pathogenesis.

Actions of inflammations on VD occurrence and development Inflammations play an important role in VD occurrence and development.^6^ A major mechanism for inflammations to cause cerebral cellular damages can be detailed as follows. Microglia are activated by inflammations firstly and the activated microglia secrete lots of IL-6 and TNF-α, etc., which further leads to neuron damages. In a study made by Yang et al.^7^ a rise of IL-6 and TNF-α expression levels may damage cognitive functions of brain cells, enabling VD to occur. Additionally, TNF-α makes the number of cellular adhesion molecules increased, resulting in basilar membrane damages and blood-brain barrier permeability rises.^8^ As a consequence, not only is brain tissue edema more serious, but more inflammatory substances are produced within the tissue. Moreover, inflammatory factors, such as IL-6 and TNF-α, has the potential to promote iN-OS production by nerve cells; and, iN-OS may further exacerbate neuronal damages. It is reported by Kim et al.^9^ that proinflammatory factors (e.g., IL and TNF-α) can bring about acetylcholine synthesis reduction within brain due to their damages to choline. Consequently, functions of the cholinergic system are impaired and eventually VD occurs. CRP is a common target that can be used to evaluate inflammatory responses. In opinions of Uemura J et al.^10^, CRP is capable of activating complement systems of the human body, causing cerebrovascular intima damages, inducing atherosclerosis, and developing cerebrovascular diseases such as cerebral infarction. It is proved in the present study that expression levels of IL-6, CRP and TNF-α proteins in VD patients are higher than those in the normal population on one hand, and positive correlated to dementia degrees on the other hand. This indicates that VD occurrence and development are associated with involvement of inflammatory factors. In addition, data of the experimental group also manifest that correlation of CRP is at the minimum for a possible reason that all cases in this group have a rather long course of disease. Because CRP increases rapidly in the acute inflammatory response, but not as much in the chronic inflammatory response.

Actions of oxidation-antioxidation imbalance on VD occurrence and development Imbalance between oxidation and anti-oxidation is an important pathogenic factor causing VD. As demonstrated by animal model tests^11^, lots of unsaturated fatty acid produced by oxidation of oxygen radicals are present in brain tissues of rats in the VD model. Such unsaturated fatty acid may lead to cerebral neuron envelope permeability changes, cellular edema and intracellular/extracellular osmotic pressure variations. Consequently, neuronal necrosis and dysfunction may occur, which further leads to a series of abnormalities in cognition, mentality and memory, etc.^12^ Clearly, oxidation-antioxidation imbalance is a major reason for VD occurrence and development. In this context, VD development and treatment effects may be predicted by evaluating and dynamically observing expression levels of anti-oxidative molecules in VD patients and their changing processes. SOD is believed to be an antioxidant substance most extensively investigated at present; decline in SOD content is mostly a sign of antioxidant capacity reduction in tissues. Specific values of TAC reveal antioxidant functions of the body; and, in most cases, they are inclined to drop under circumstances of inflammatory responses. In terms of CAT, it is capable of removing oxygen radicals produced during a catalytic reaction with SOD.^13^ Anti-oxidative molecules mentioned above play a protective role in inflammatory responses and ischemic injuries of the body. In addition, GST is able to eliminate lipid peroxides. Present in intestinal tracts and liver tissues generally, GR can turn oxidized glutathione into a reduced form by using reduced NAD based on a catalytic reaction.^14^ In the present study, expression levels of SOD, TAC and CAT show an obvious tendency of decline with increases in severity of illness. This manifests that imbalance between oxidation and anti-oxidation takes place in VD patients.

Regarding GST and GR, no significant variations are found. Possible reasons may be as follows:

1. Sample size of the present study is rather small.
2. Inclusion criteria adopted for the present study are rather strict, so that some patients are excluded.

### Limitations of this study

This study is a retrospective study, and the results of data analysis are not as convincing as those of prospective studies. And our sample size is not large enough. In future studies, we will try to demonstrate the effect of inflammatory response and antioxidant response on vascular dementia through animal experiments and cell experiments.

## CONCLUSIONS

1. VD patients show a significantly higher level of serum inflammatory factors than the normal population does, while content of anti-oxidative molecules in the former is below that of the latter.
2. Considering that TNF-a, IL-6, SOD, TCA and CAT are significantly correlated with dementia degrees, they can be used as risk factors of VD prediction and measures of efficacy evaluation.

To sum up, content of some inflammatory factors and anti-oxidative molecules in VD patients changes more obviously than the normal population does. In addition, their content forms a significant correlation to dementia degree. On this basis, it is concluded that dynamic monitoring on inflammatory factors and anti-oxidative molecules has certain significance for severity evaluation or therapeutic effect judgment among VD patients. Featured with simple clinical operations and low cost, this is a method worth applying.

### Authors’ Contributions:

**FQandYH** designed this study and prepared this manuscript, and are responsible and accountable for the accuracy or integrity of the work.

**JG** collected and analyzed clinical data. **YL** significantly revised this manuscript.

## References

[1] Smith EE (2017). Clinical presentations and epidemiology of vascular dementia. Clin Sci (Lond).

[2] Perani D, Cerami C, Caminiti SP, Santangelo R, Coppi E, Ferrari L (2016). Erratum to:Cross-validation of biomarkers for the early differential diagnosis and prognosis of dementia in a clinical setting. Eur J Nucl Med Mol Imaging.

[3] Iadecola C (2013). The pathobiology of vascular dementia. Neuron.

[4] O'Brien JT, Thomas A (2015). Vascular dementia. Lancet.

[5] Calabrese V, Giordano J, Signorile A, Laura Ontario M, Castorina S, De Pasquale C (2016). Major pathogenic mechanisms in vascular dementia:Roles of cellular stress response and hormesis in neuroprotection. J Neurosci Res.

[6] Campo GM, Avenoso A, Campo S, D'Ascola A, Traina P, Rugolo CA (2010). Differential effect of molecular mass hyaluronan on lipopolysaccharide-induced damage in chondrocytes. Innate Immun.

[7] Yang EJ, Cai M, Lee JH (2016). Neuroprotective Effects of Electroacupuncture on an Animal Model of Bilateral Common Carotid Artery Occlusion. Mol Neurobiol.

[8] Wang F, Zou Z, Gong Y, Yuan D, Chen X, Sun T (2017). Regulation of Human Brain Microvascular Endothelial Cell Adhesion and Barrier Functions by Memantine. J Mol Neurosci.

[9] Kim MS, Bang JH, Lee J, Han JS, Kang HW, Jeon WK (2016). Fructus mume Ethanol Extract Prevents Inflammation and Normalizes the Septohippocampal Cholinergic System in a Rat Model of Chronic Cerebral Hypoperfusion. J Med Food.

[10] Uemura J, Ohta M, Yamashita S, Yagita Y, Inoue T (2020). C-reactive Protein is A Predictor of Deterioration of Acute Internal Carotid Artery M1 Qcclusion Following Recanalization. J Stroke Cerebrovasc Dis.

[11] Kono K, Shintani A, Yoshimura R, Okada H, Tanaka Y, Fujimoto T (2013). Triple antiplatelet therapy with addition of cilostazol to aspirin and clopidogrel for Y-stent-assisted coil embolization of cerebral aneurysms. Acta Neurochir (Wien).

[12] Nakagawa I, Wada T, Park HS, Nishimura F, Yamada S, Nakagawa H (2014). Platelet inhibition by adjunctive cilostazol suppresses the frequency of cerebral ischemic lesions after carotid artery stenting in patients with carotid artery stenosis. J Vasc Surg.

[13] Arazi H, Taati B, Rafati Sajedi F, Suzuki K (2019). Salivary Antioxidants Status Following Progressive Aerobic Exercise:What Are the Differences between Waterpipe Smokers and Non-Smokers?. Antioxidants (Basel).

[14] Arazi H, Simaei E, Taati B (2016). Comparison of responses of salivary antioxidant markers to exhaustive aerobic exercise in smoker and non-smoker young girls. J Sports Med Phys Fitness.

